# Typhus Fever and Difficulties in Diagnosis

**Published:** 1894-06-02

**Authors:** 


### Typhus Fever and Difficulties in Diagnosis.

London is never entirely free from typhus fever, but of late years the mortality caused by it has dwindled to such small dimensions, that one has almost ceased to look upon it as a public danger of any importance. The older men amongst us, however, can well remember how serious an affair it used to be, and how greatly it added to the perils of practice; and the experience even of late years in London, and that quite recently gained in Liverpool, shows that whatever may be the cause of the lessened prevalence of the malady during recent years, it is not from any change in the disease itself; the old enemy is just the same as ever, and when it gains a footing spreads in the same way as it used to do.

Its early diagnosis then becomes a matter of very great importance, for it is by early removal to hospital and early disinfection of premises, that we can best prevent the disease from becoming epidemic.

The deaths from typhus registered in London during recent years have been as follows :—

| | Deaths. | | Deaths. |
|---|---|---|---|
| 1881 | 92 | 1888 | 9 |
| 1882 | 53 | 1889 | 16 |
| 1883 | 55 | 1890 | 10 |
| 1884 | 32 | 1891 | 11 |
| 1885 | 28 | 1892 | 11 |
| 1886 | 13 | 1893 | 5 |
| 1887 | 19 | | |

From this the great diminution of the disease is obvious, and, in fact, it is now a fever of such rarity, that there are plenty of medical men in practice who have hardly ever, or even perhaps have never, come across it.

Herein lies a very considerable danger, for even to those who have seen many cases the diagnosis in the early stages is by no means easy, and it is much to be feared that many cases are missed, overlooked partly from want of experience, but partly also in consequence of all thought of typhus being out of men's minds. At any rate, whatever the cause may be, it seems a fact that typhus fever often fails to be identified. Dr. Hope, medical officer of health for Liverpool, has stated that in the recent epidemic in that town a large proportion of the cases were not correctly diagnosed, and that out of twenty-five cases of typhus fever removed to hospital during the week ending February 24th not one was notified as such.

This is a serious condition of affairs, and shows how necessary it is constantly to bear in mind, not for our excuse but for our guidance, how frequently typhus is either mistaken for some other form of fever or looked upon as a mere typhoid condition occurring in the course of some non-zymotic febrile ailment, a common enough condition in overcrowded and insanitary dwellings.

In relation to this point Dr. Shirley Murphy presented an interesting report to the London County Council in December, 1891, which is worth consideration as showing how long an epidemic may go on unrecognized even by good men.

In October, 1889, attention was called to a disease characterised by pneumonic symptoms and delirium, which had been prevalent in a narrow ill-ventilated court, going by the name of Sard's Rents, since January, 1888. One patient had been removed to a public institution and died there, his death being registered as pneumonia; another had died in a general hospital from what was recorded as bronchitis and emphysema, but what, from later inquiries, probably was typhus fever. On first visiting the place the only cases seen were some children who were recovering, and no definite diagnosis could be arrived at, but a few days later other persons sickened and were carefully examined both by Dr. Murphy and Dr. Wightwick The disease evidently was not pneumonia, nor was there any eruption to class it as typhus, but the general appearance of the patients, together with the behaviour of the disease in the court, raised suspicions as to its nature, and an inspection of the notes of the case which had died in hospital strengthened the impression that the patients were suffering from typhus fever. It was therefore arranged that all subsequent cases should be removed to hospital, where the disease was then definitely recognised as typhus fever. From that time every person attacked was removed and the houses disinfected. The epidemic speedily terminated.

The history of a group of cases occurring in Holborn, together with its probable interlinking with other groups, is full of interest. The house was occupied by two families, S. and H. The father of the S. family had been out of regular work, and had had temporary employment at an old clothes shop in an adjoining street. He was the first to be taken ill; three weeks after his wife suffered from an illness which terminated fatally a few days later. An inquest into the cause of the wife's death was held, the verdict being that she died of fatty degeneration of the heart. Two or three days after this woman's death one of her sons sickened. A second son was attacked two or three days later, his illness being followed by that of five of his sisters. One of these girls was in service in the Strand, the girl working there in the day, and attending her family at night. Her master and mistress were, on October 15th, certified to have typhoid fever, and subsequently her master died. On October 28th and 29th two members of the H. family, who lived in the same house in Holborn as the S. family, were also certified to have typhoid; and, lastly, another member of this family was on November 3rd certified to have typhus. Inquiry made by Dr. Gibbon led to the conclusion that all these people suffered from typhus, and this opinion was shared by Dr. Cook, the resident medical officer of the hospital, so far as concerned those cases (eight in number) which came under his observation.

It is clear from these cases, and from many others to which we could refer, notably the history of the outbreak which occurred last year in the gaols of Paris and in the northern parts of France, that whatever the cause may be. practitioners often fail to recognise typhus fever. Years ago it was not so. There were difficult cases then as now, and there were mistakes; but at any rate the question was mentally discussed, and the occurrence of grouping together of fever, headache, and drowsiness, always raised at once in men's minds the possibility of typhus. It is clear that it behoves us constantly to be on the look out for this most insidious disease, for unless the possibility of its occurrence be continually borne in mind, all experience shows how easy it is to make errors of diagnosis.

